# VPS35 Deficiency Markedly Reduces the Proliferation of HEK293 Cells

**DOI:** 10.3390/genes17020177

**Published:** 2026-01-31

**Authors:** Sujin Lee, Soojin Park, Hyewon Bang, Sun-Uk Kim, Young-Ho Park, Gabbine Wee, Unbin Chae, Ekyune Kim

**Affiliations:** 1 College of Pharmacy, Catholic University of Daegu, Gyeongsan-si 38430, Gyeongbuk, Republic of Korea; 2 Futuristic Animal Resource & Research Center, Korea Research Institute of Bioscience and Biotechnology (KRIBB), Cheongju-si 28116, Chungcheongbuk-do, Republic of Korea; 3 Laboratory Animal Center, Daegu-Gyeongbuk Medical Innovation Foundation, Daegu 41061, Gyeongbuk, Republic of Korea

**Keywords:** VPS35, retromer complex, HEK293 cells, mitochondrial dynamics, cell proliferation, endosomal trafficking

## Abstract

**Background/Objectives:** The retromer protein complex is involved in various physiological processes, especially endosomal trafficking, and its dysregulation has been linked to Alzheimer’s disease and Parkinson’s disease, as well as VPS35 knockout (KO), causing early embryonic lethality. We aimed to investigate the cellular consequences of VPS35 deficiency. **Methods:** To investigate the effects of VPS35 loss, we used CRISPR/Cas9 to generate VPS35 KO human embryonic kidney 293 (HEK293) cells. We analyzed changes in retromer component expression, cell proliferation, apoptosis, and mitochondrial dynamics using Western blotting, terminal deoxynucleotidyl transferase dUTP nick end labeling (TUNEL) assay, and confocal microscopy. **Results:** VPS35 KO led to a significant reduction in cell proliferation and decreased expression of VPS29 and VPS26, both essential for retromer complex assembly. Consequently, retromer formation was impaired. Compared to control cells, KO cells exhibited elevated levels of cleaved caspase-3, poly(ADP-ribose) polymerase, cytochrome C, and p21, while the expression of Ki-67, CDK4, and cyclin D was reduced. Additionally, VPS35 deletion also promoted mitochondrial fragmentation, associated with increased expression of mitochondrial fission-related proteins. Finally, the rescue experiment using the human VPS35 gene confirmed that the recovery of VPS35 not only led to the recovery of the essential elements constituting the retromer but also the recovery of molecules related to the cell cycle, restoring cell death to a normal level. **Conclusions:** These findings suggest that VPS35 plays a critical role in cell growth and survival by modulating apoptosis, mitochondrial dynamics, and cell cycle progression.

## 1. Introduction

To maintain cell homeostasis, essential proteins must be synthesized and transported to their proper location based on the cell’s genetic information, while used molecules are directed to lysosomes for degradation and recycling [1,2,3]. Particularly, molecules present in the cell membrane undergo cycles of expression and disappearance depending on the external environment, and cargo proteins are known to play an important role in this process [4,5]. The vacuolar protein sorting (VPS) family of proteins was first discovered in yeast and has been shown to participate in directing proteins to their appropriate cellular destinations [6,7,8]. VPS proteins are therefore reported to play vital roles in various biological processes, including early embryonic development [9,10], vacuolar hydrolase transport [11], and transcytosis of cell membrane proteins [12]. Besides these functions in yeast, VPS proteins are major constituents of the mammalian retromer complex, which was first characterized in mice. The retromer, a complex composed of VPS26, VPS29, and VPS35, plays a central role in the retrieval of specific cargo proteins from endosomes to the trans-Golgi network [13,14,15]. This complex is indispensable for the efficient recycling of transmembrane receptors such as the cation-independent mannose-6-phosphate receptor (CI-M6PR) and epidermal growth factor receptor (EGFR), thereby maintaining intracellular protein homeostasis [16]. Depletion of VPS35 disrupts autophagy and endolysosomal homeostasis by impairing the recycling of autophagy regulators, resulting in the accumulation of autophagosomes and lysosomal dysfunction [17]. A deficiency in retromer function causes transport defects of GLUT1 and Wnt receptors, endosomal trapping abnormalities, increased expression of endoplasmic reticulum stress-related genes, impaired cell differentiation, and reduced cell survival [18,19,20]. Furthermore, retromer dysfunction causes the accumulation of proteins such as α-synuclein, APP, and tau in brain tissue, leading to neurodegenerative disorders such as Parkinson’s and Alzheimer’s diseases, as well as abnormalities in mitochondrial structure and division [21,22,23]. Although the retromer complex has been primarily investigated in the context of neurodegenerative diseases [21,22,23], the observation that deletion of VPS26 or VPS35 causes embryonic lethality in mice indicates that retromer function is also essential for maintaining fundamental cellular homeostasis. [24].

In the current study, we sought to elucidate the importance of VPS35 in maintaining cell homeostasis by eliminating VPS35 from HEK293 cells. Collectively, our findings suggest that reduced retromer function inhibits cell proliferation and that modulation of this pathway may influence factors regulating the proliferative process. Therefore, therapeutic strategies aimed at enhancing retromer expression should be carefully considered in contexts involving apoptosis, decreased cell proliferation, and mitochondrial abnormalities.

## 2. Materials and Methods

### 2.1. Cell Culture

The human embryonic kidney 293 (HEK293) cell line was obtained from the Korean Cell Line Bank and cultured in Dulbecco’s modified Eagle’s medium (Thermo Fisher Scientific, Langenselbold, Germany; 30966-021) supplemented with 10% fetal bovine serum (Thermo Fisher, Langenselbold, Germany; A31608-01). Cultivation was kept under sterile conditions and incubated as adjerent monolayer at 37 °C in an atmosphere containing 5% CO_2_ and 80% relative humidity. For subculturing, cells were harvested using 0.25% trypsin-ethylene diaminetetraacetic acid (EDTA) (Thermo Fisher, Langenselbold, Germany; 25200-072) [24].

### 2.2. Establishment and Characterization of the VPS35-Deficient HEK293 Cell Line

VPS35 knockout was achieved using the CRISPR/Cas9 genome-editing system targeting exon 4 of the VPS35 gene (chromosome 16, 17 exons) [25]. Guide RNAs (gRNAs) were designed, synthesized, and ligated into a pX330 plasmid to target the DNA sequence of exon 4 in the VPS35 gene. The plasmid containing both the gRNAs and Cas9 was transfected into HEK293 cells withLipofectamine^®^ 3000 (Thermo Fisher, Langenselbold, Germany; #L3000015). After 24 h, single-cell cloning was performed in 96-well plates Genomic DNA was amplified by PCR using a pair of forward and reverse primers (P1: 5′ ATGTTTATCCAGTGGGTGACACAG-3′ and P2: 5′-CCAGTCTATAACAGCAAGTCCTTC-3′), and mutations were verified by Sanger sequencing.

### 2.3. RT-PCR Analysis

Total RNA was extracted from *Vps35^+/+^* and *Vps35^−/−^* HEK293 cells using Isogen, as described previously [12]. Briefly, cDNA was synthesized from 5 μg of total RNA with SuperScript III First-Strand Synthesis System (Invitrogen, Carlsbad, CA, USA). PCR amplification was performed using Ex Taq DNA polymerase (Takara, Shiga, Japan) ‘with the following primer sets: *Vps35*: 5′-CACAGTTGGAGTTGTATATGTCAA-3′ (forward), 5′-AGCAATGATTATGTTCTTCACA-3′ (reverse), *Vps26*: 5′-ATGACAAGAGTAATACTCATGA-3′ (forward), 5′-TTACTGGTGCACCATCCATTAT-3′ (reverse), Vps29: 5′-ATGAGCAGGTGTGCTCTCAGAGG-3′ (forward), 5′-CAACACAAATGATGGAATAATG-3′ (reverse), *GAPDH*: 5′-AGATTGTCAGCAATCATCCTG-3′ (forward), 5′-TGCTTCACCACCTTCTTGATGT-3′ (reverse). The PCR cycling protocol was 94 °C for 60 s, 60 °C for 60 s, and 72 °C for 60 s for 35 cycles. Amplified DNA products were analyzed using 1.5% agarose gel electrophoresis as described previously [13].

### 2.4. Preparation of Protein

HEK293 cells were harvested and washed with phosphate-buffered saline (PBS) by centrifugation at 800× *g* for 10 min. Proteins were extracted using 20 mM Tris–HCl (pH 7.4) containing 1% Triton X-100, 0.15 M NaCl, and 1% protease inhibitor cocktail (Roche, Indianapolis, IN, USA) on ice for 2 h. The lysates were clarified by centrifugation at 13,000× *g* for 10 min at 4 °C, and protein concentration was determined using a Coomassie protein assay kit (Pierce, Rockford, IL, USA) [26].

### 2.5. Antibodies

Polyclonal anti-rabbit VPS26 and polyclonal anti-rabbit VPS35 antibodies were prepared as previously reported [13]. The following primary antibodies were used: anti-caspase-3 (9662S; 1:3000), anti-poly(ADP-ribose) polymerase (PARP; 9542S; 1:3000), anticytochrome C (11940S; 1:1000), anti-CDK4 (12790S; 1:2000), anti-cyclinD1 (2978S; 1:3000), anti-Drp1 (8570S; 1:3000), and anti-pDrp1 (S616) (3455S; 1:1000) (all from Cell Signaling Technology, Danvers, MA, USA); anti-Ki-67 (ab15580; 1:1000; Abcam, Boston, MA, USA); anti-Mfn2 (sc-515647; 1:1000; Santa Cruz Biotechnology, Dallas, TX, USA); and anti-GAPDH (LF-PA0018; 1:3000; AbFrontier, Seoul, Republic of Korea).

### 2.6. Western Blot Analysis

50 µg of protein were resolved on 8.5–12.5% SDS-PAGE gels and transferred onto PVDF membrane (Immobilon-P; Millipore, Burlington, MA,USA). Membranes were then blocked with 2% skim milk in 20 mM Tris-HCl, pH 7.4; 0.3M NaCl,; 0.1% Tween-20), and incubated with the primary antibodies listed above for 2 h at room temperature, followed by with horseradish peroxidase-conjugated secondary antibodies. Protein bands were visualized using an enhanced chemiluminescence detection kit (ELPIS-BIOTECH, Daejeon, Republic of Korea) and visualized using the Odyssey Infrared Imaging System (LI-COR Biosciences, Lincoln, NE, USA). Band intensities were quantified using the ImageJ software (version 1.54p; NIH, Bethesda, MD, USA) [27].

### 2.7. Imaging Mitochondrial Morphology

HEK293 control and VPS35 KO cells were seeded onto Lab-Tek™ II chamber slides (Thermo Fisher Scientific, Carlsbad, CA, USA) and cultured for 24 h. Cells were incubated for 45 min at 37 °C in pre-warmed Dulbecco’s modified Eagle’s medium (DMEM) containing the MitoTracker probe (Thermo Fisher Scientific). After incubation, cells were washed twice with PBS and fixed with 4% paraformaldehyde (Sigma-Aldrich, St. Louis, MO, USA) for 1 h. Following three PBS washes, coverslips were mounted on slides using the VECTASHIELD mounting medium (VECTOR Laboratories, Newark, CA, USA). Mitochondrial morphology was visualized with LSM 710 confocal microscope (Carl Zeiss, Oberkochen, Germany) equipped with a Plan-Apochromat 100×/1.40 oil DIC M27 objective. Images were processed with ZEN 2009 Light Edition (Carl Zeiss, Oberkochen, Germany).

### 2.8. Rescue Experiment

The VPS35-defecient HEK293 cells were cultured in DMEM supplemented 10% heat-inactivated FBS, 10 IU/mL penicillin, and 10 mg/mL streptomycin. The cells were plated on poly-D-lysine coated 60 mm dishes. The KO cells were transfected with the pcDNA3.1-VPS35 gene expression vector using Lipofectamine 2000 (Invitrogen, Carlsbad, CA, USA) according to the manufacturer’s instructions and previousreport [13]. Cells were cultured for 48 h, and transfected cells were then selected in G418-containing medium to establish stable expression.

### 2.9. Statistical Analysis

All quantative data are shown as the mean ± SD from three independent experiments (*n* = 3). Statistical significance was evaluated by one-way or two-way ANOVA using GraphPad Prism (version 8, GraphPad Prism Software, San Diego, CA, USA); *p*-values of <0.01 and <0.001 are indicated in the figures using two and three asterisks, respectively.

## 3. Results and Discussion

**Figure 1:**
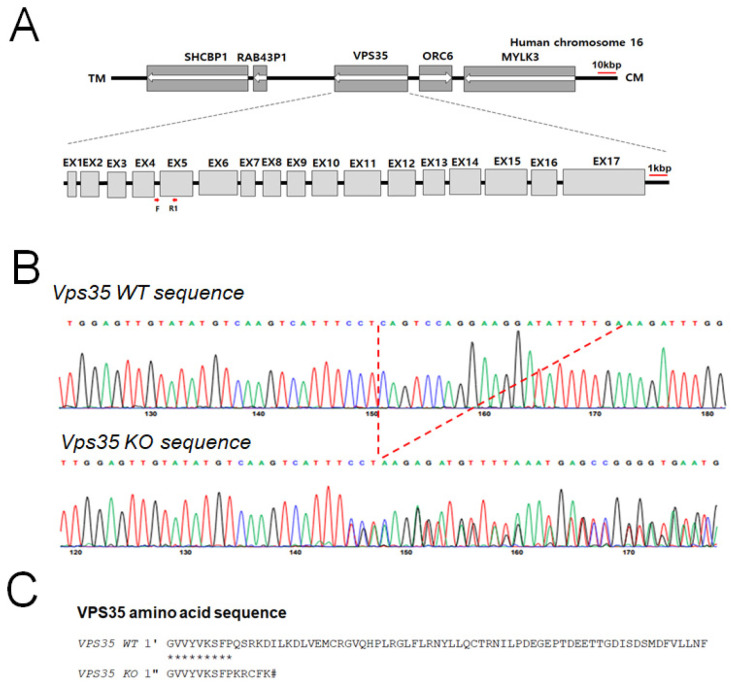
Generation of VPS35-deficient HEK293 cells: (**A**) Schematic representation of human chromosome 16 showing the VPS35 locus. A genomic region encompassing the coding sequence was deleted using the CRISPR/Cas9 system. (**B**) Genomic sequence of the CRISPR/Cas9 target site in VPS35. Letters within the dotted lines indicate the deleted bases. (**C**) Alignment of the predicted amino acid sequences of wild-type (WT) and VPS35-knockout (KO) alleles. Asterisks (*) indicate amino acids conserved between the two sequences, and the hash symbol (#) denotes the stop codon introduced by the deletion.

**Figure 2:**
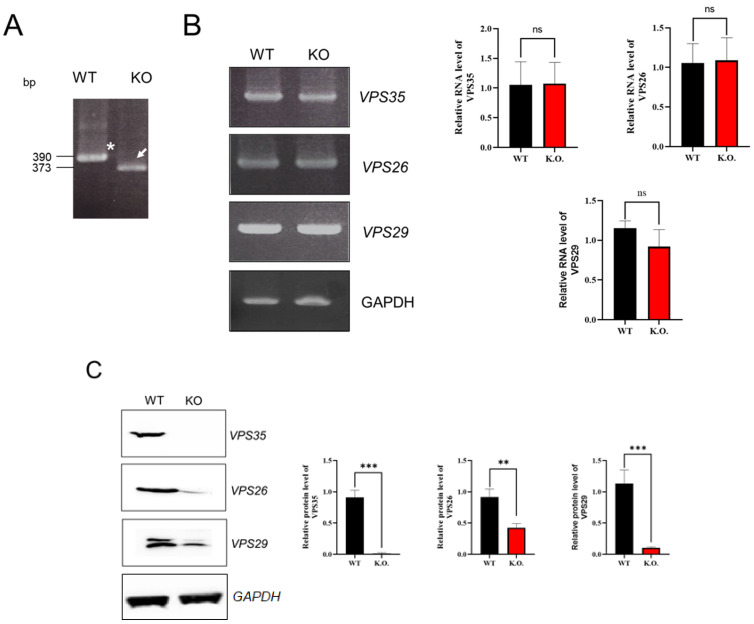
Characterization of VPS35 KO HEK293 cells: (**A**) Genomic DNA PCR results from WT and VPS35 KO cells. An asterisk (*) indicates a WT band, and arrows (→) indicate the VPS35 KO allele. (**B**) RT-PCR analysis of VPS35, VPS26, VPS29, and GAPDH using complementary DNA from WT and KO cells. (**C**) Western blot analysis of protein lysates from WT and KO cells. Proteins were extracted using 1% Triton X-100 and separated via SDS-PAGE. Data are representative of three independent experiments (*n* = 3). *** *p* < 0.001, ** *p* < 0.01, ns; not significant.

We evaluated retromer protein expression following VPS35 KO. Previous studies have investigated retromer function by modulating gene expression [14]; however, no experiments have utilized CRISPR/Cas9 to knock out retromer molecules. In this study, we aimed to elucidate the cellular consequences of VPS35 deficiency, as VPS35 is a core component of the retromer complex. Notably, complete VPS35 KO in mice results in embryonic lethality [24]. To elucidate the functional role of VPS35, we knocked out VPS35 in the HEK293 cells using CRISPR/Cas9-mediated genome editing (Figure 1A). The genotypes of WT (VPS35^+/+^) and homozygous KO (VPS35^−/−^) cells were confirmed via PCR analysis of genomic DNA. CRISPR/Cas9 mediation successfully eliminated 17 bases within exon 5 of VPS35 (Figure 1A,B and 2A). Sequencing analysis revealed that this deletion introduced a premature stop codon at the 42nd amino acid position of VPS35 (Figure 1C).

Moreover, the immunoreactive 95 kDa protein band corresponding to VPS35 was absent in Western blots of protein extracts derived from VPS35 KO HEK293 cells. Notably, the expression of VPS26 and VPS29 retromer components was downregulated by approximately 50% according to Western blot analysis (Figure 2C), although the *VPS26*, *VPS29*, and mRNA levels in VPS35 KO HEK293 cells remained unchanged (Figure 2B). Interestingly, the partial deletion of *VPS35* mRNA not only expressed at normal levels but also had no effect on *VPS26* and *VPS29* mRNA expression. These results indicate that the lack of VPS35 leads to malformation of the retromer complex. Consequently, the structural instability of the retromer complex caused by VPS35 deficiency reduces the stability and expression levels of its constituent proteins.

**Figure 3:**
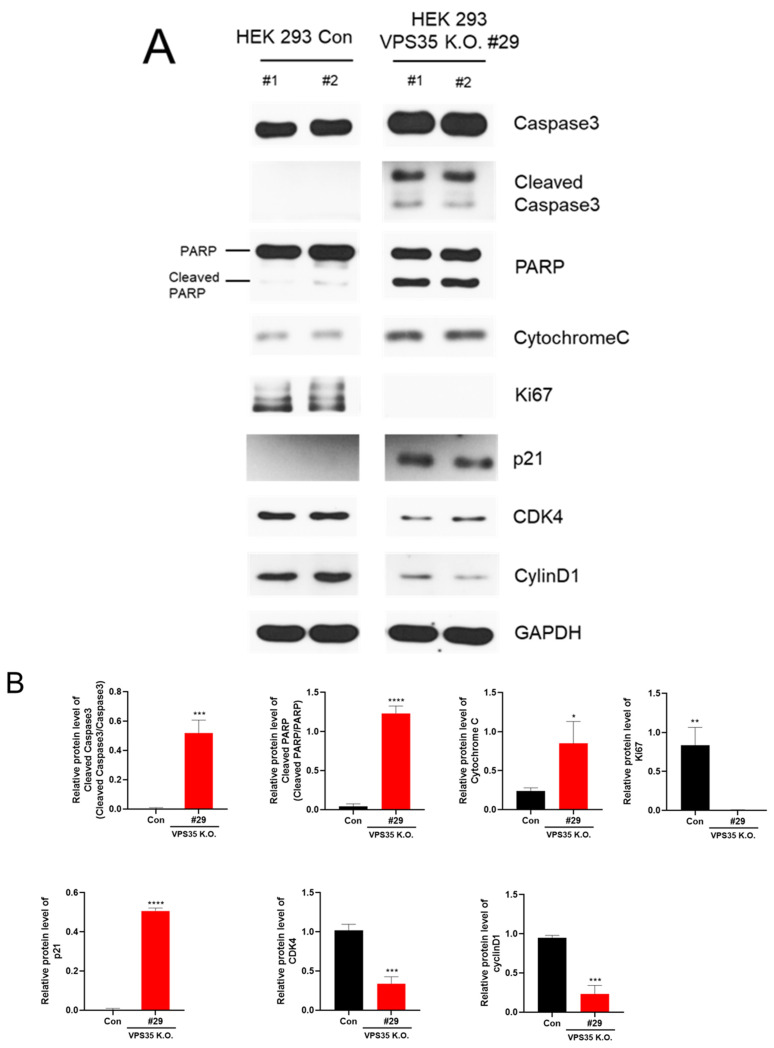
VPS35 deficiency leads to activation of apoptotic signaling and cycle-related proteins. (**A**) Endogenous protein levels in VPS35 KO HEK293 cells were analyzed via Western blotting using antibodies against the indicated proteins. (**B**) Protein band intensities were quantified and compared with scramble control cells. Data are representative of three independent experiments (*n* = 3). **** *p* < 0.0001, *** *p* < 0.001, ** *p* < 0.01, * *p* < 0.05.

Given that VPS35 is an essential component of the retromer complex, whole-body KO of VPS35 is embryonic lethal [24]. However, the mechanisms underlying VPS35 deletion-induced lethality remain unclear. Cells regulate their fate through the selective expression of proteins that determine whether they undergo proliferation or apoptosis. In VPS35-deficient cell lines, a pronounced impairment of cell proliferation has been observed, suggesting that VPS35 is a critical regulator of cell growth.To investigate the underlying molecular mechanisms, we performed Western blot analysis of proteins involved in apoptosis and the cell cycle. Western blot analysis showed a significant increase in caspase-3 expression in VPS35 KO cells compared to that in WT control cells. Importantly, the level of cleaved caspase-3, the activated form that serves as a well-established hallmark of apoptosis, was markedly elevated in VPS35 KO cells. In canonical apoptotic pathways, caspase-8 and caspase-9 initiate the proteolytic activation of caspase-3, generating its cleaved form, which subsequently induces cell death by processing downstream substrates such as PARP [28]. Consistent with this mechanistic cascade, PARP cleavage was further assessed in VPS35 KO cells, revealing a pronounced increase in cleaved PARP levels and reinforcing the activation of apoptotic signaling in the absence of VPS35 (Figure 3A,B).

**Figure 4:**
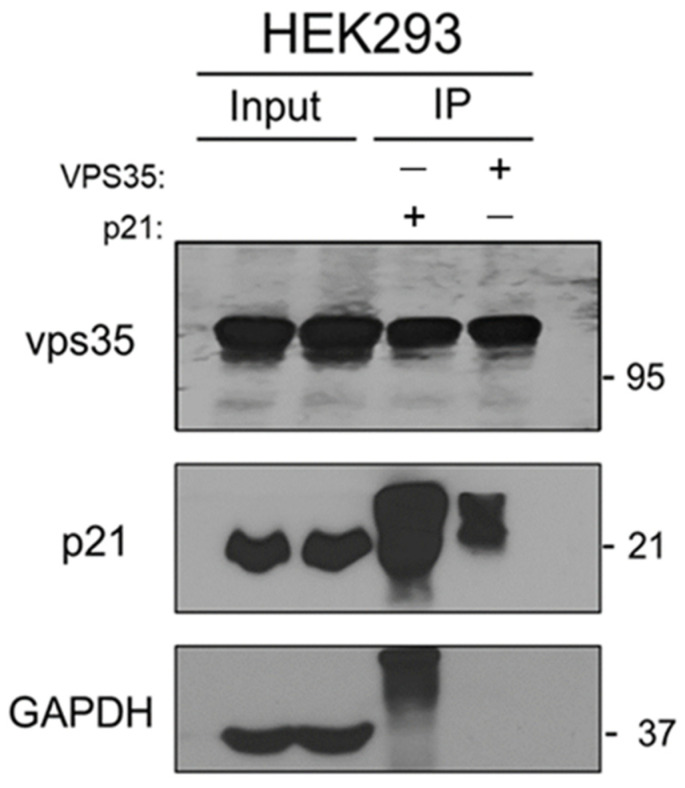
Immunoprecipitation assay with HEK293 cell extracts. The protein extracts from HEK293 were immunoprecipitated with anti-Vps35 and p21 antibodies and subjected to Western blot analysis using the antibodies indicated on the left side of the image.

To further characterize apoptotic events associated with VPS35 deficiency, cytochrome C expression was examined in HEK293 cells. Cytochrome C, a mitochondrial intermembrane protein, is released into the cytosol upon apoptotic stimulation, where it promotes caspase-3 activation through the apoptosome pathway [29]. This release is facilitated by apoptotic signals that elevate intracellular calcium levels and trigger opening of the mitochondrial permeability transition pore [30]. Consistent with this intrinsic apoptotic mechanism, Western blot analysis revealed a significant increase in cytochrome c levels in VPS35 KO cells compared with WT controls (Figure 3), supporting a role for VPS35 in regulating mitochondrial-mediated apoptosis. Moreover, the expression of Ki-67, a well-established proliferation marker [31], was markedly reduced in VPS35-deficient cells, indicating a pronounced impairment of cell growth (Figure 3). These findings suggest that VPS35 deficiency has a considerable impact on cell cycle regulatory pathways. Among critical regulators of the cell cycle, p21 has been well characterized as a cyclin-dependent kinase (CDK) inhibitor that negatively affects cell viability [32]. Therefore, we examined p21 expression to elucidate why VPS35-deficient cells exhibit impaired cell proliferation. Western blot analysis revealed a marked upregulation of p21 levels in VPS35 KO cells (Figure 3A,B). Next, we evaluated the influence of p21 on downstream cell cycle-related proteins, including CDK4 and cyclin D1. As expected, both proteins were significantly downregulated in the KO cells, suggesting that the elevated p21 levels can suppress CDK4 activity, thereby inducing cell cycle arrest at the G1 and G2 phases. Although the direct mechanism underlying p21 upregulation in VPS35-deficient cells remains unclear, VPS35 participates in the transport of cytoplasmic proteins to lysosomes for degradation, thereby contributing to cellular homeostasis. The absence of VPS35 likely impairs protein trafficking and degradation, leading to cellular stress that may account for the elevated expression of p21. Supporting this hypothesis, immunoprecipitation analysis indicated a functional interaction between VPS35 and p21 (Figure 4). Collectively, these findings suggest that VPS35 normally suppresses p21 expression, thereby promoting cell cycle progression, whereas VPS35 deficiency stabilizes p21, leading to its accumulation, cell cycle arrest, and enhanced apoptosis.

**Figure 5:**
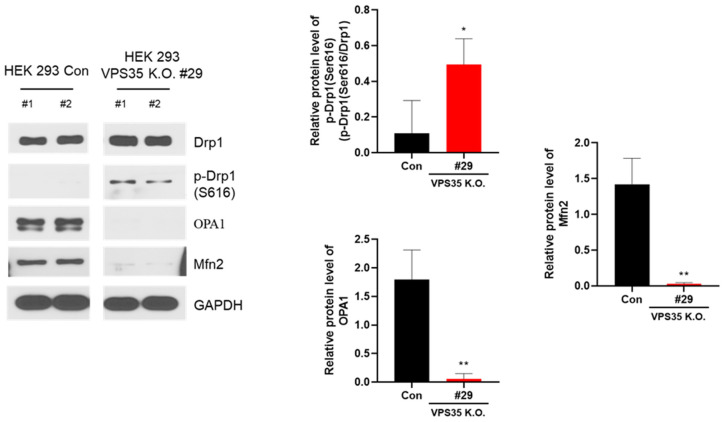
VPS35 deficiency alters mitochondrial dynamics in HEK293 cells. Western blot analysis of total and phosphorylated Drp1, OPA1, Mfn1, and Mfn2 expression in wild-type (WT) and VPS35 knockout (KO) HEK293 cells. Protein band intensities were quantified and compared with scramble control cells. Data are representative of three independent experiments (*n* = 3). ** *p* < 0.01, * *p* < 0.05.

To maintain cellular homeostasis, cells must be continuously supplied with ATP, and mutations in genes involved in mitochondrial maintenance are closely associated with cell death. In particular, studies have shown that the regulation of mitochondrial dynamics influences the expression of Drp1 protein, which is modulated by CDK and protein kinase A [33,34]. Therefore, we examined changes in the expression of Drp1, optic atrophy 1 (OPA1), and Mfn2 in VPS35 KO cells. Total Drp1 expression remained unchanged, whereas the phosphorylation of Drp1 was markedly increased in the KO cells (Figure 5). Phosphorylation of Drp1 at Ser616, mediated by CDK1/cyclin B, promotes mitochondrial fission [35]. Mitochondrial fusion is a process in which two or more mitochondria fuse to form a single large mitochondrion. Mitochondria are longer when cell division occurs. In particular, the significant reduction in OPA1 and Mfn2 in VPS35 KO cells (Figure 5) indicates decreased mitochondrial activity, as both proteins play pivotal roles in outer mitochondrial membrane (OMM) fusion and mediate mitochondria–ER crosstalk, thereby facilitating calcium transfer from the ER to mitochondria [36].

**Figure 6:**
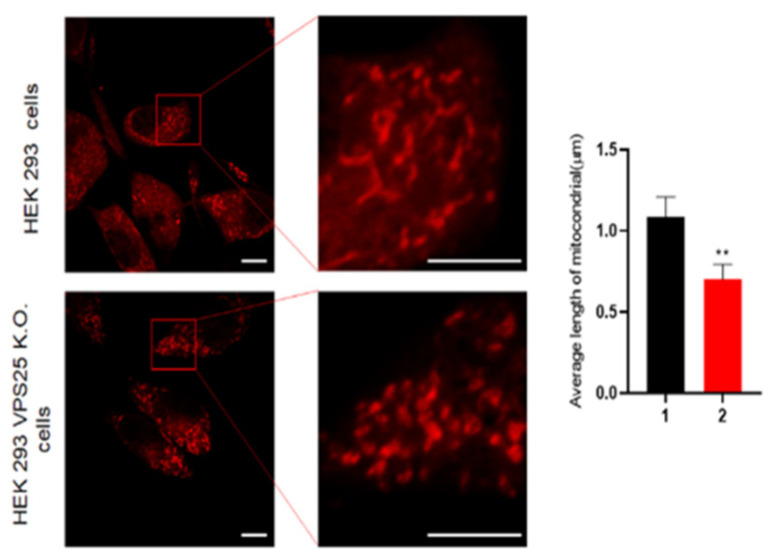
A change in mitochondrial morphology. A change in the mitochondrial morphology following control and VPS35 knockout 293 cells was observed by confocal microscopy. The right panel shows a higher magnification of the image in the square in the left panel. The scale bar represents 5 μm. ** *p* < 0.01.

**Figure 7:**
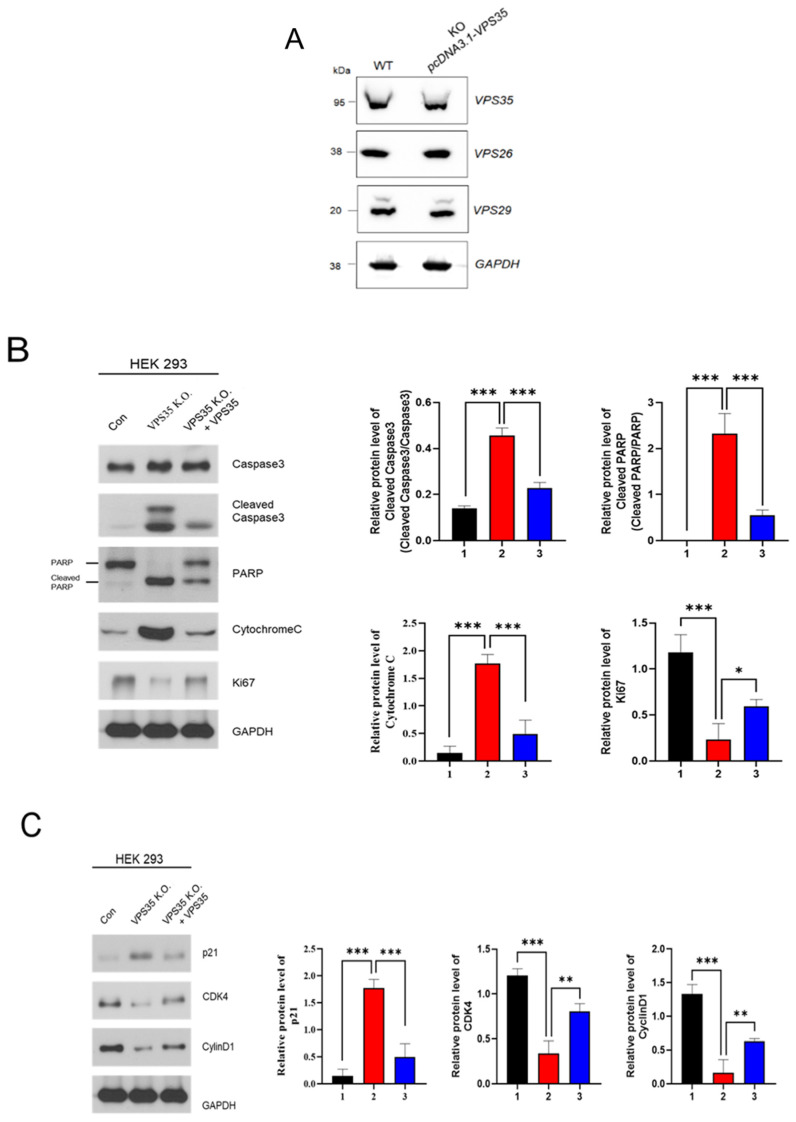

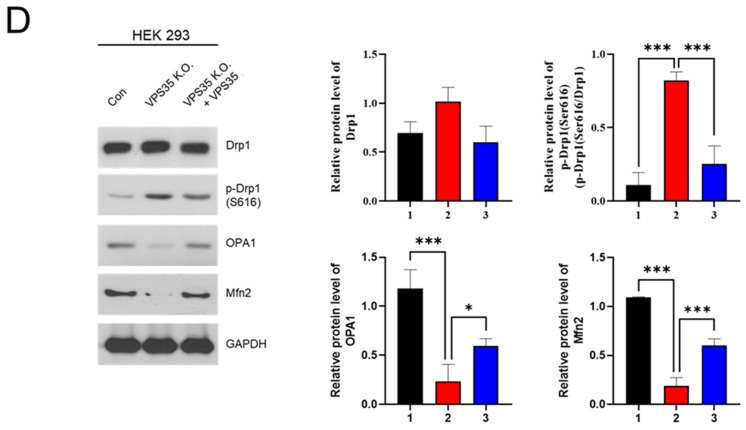
Result of the VPS35 gene rescue experiment: (**A**) The protein extracts were obtained after inserting the VPS35 gene into the KO cell, and the proteins were compared between the two groups using Western blot. (**B**) Molecules for the activation of apoptotic signaling. (**C**) Rescue of cell cycle-related protein expression. (**D**) The results of phosphorylation of Drp1, which is related to mitochondrial fission, and the expression of OPA1 and Mfn2, which are related to fusion. 1, HEK293 cells; 2, VPS35 KO HEK293 cells; 3, *VPS35* in VPS35 KO HEK 293 cells. *** *p* < 0.001, ** *p* < 0.01, * *p* < 0.05.

By contrast, mitochondrial fission is a process in which one mitochondrion is divided into two mitochondria, which occurs during the removal of damaged mitochondria and the release of cytochrome C in apoptosis [37]. We conducted a mitochondrial fragmentation assay to confirm mitochondrial dynamics (Figure 6). As expected, many mitochondrial segments occurred in the VPS35 KO cell line. To confirm whether the VPS35 KO HEK293 cell line is recovered by the VPS35 gene, a pcDNA3.1 vector containing the VPS35 was used on the KO cell line (Figure 7). As shown in Figure 7, stable expression of VPS35 in the KO cell line not only restored the expression levels of retromer components but also improved cell proliferation compared to that in KO cells. In detail, our data show that not only VPS26 and VPS29, the essential components constituting the retromer, recovered to normal levels, but also the molecules involved in apoptosis, the cell cycle, and mitochondrial fission, as shown in Figure 7.

Collectively, these findings suggest that the absence of VPS35 promotes Drp1 phosphorylation, likely owing to cellular stress arising from impaired cytoplasmic protein transport. This enhanced phosphorylation of Drp1 may, in turn, promote apoptosis. In contrast, OPA1, a GTPase localized to the inner mitochondrial membrane, plays a critical role in mitochondrial fusion and the maintenance of cristae structure. Therefore, sustained OPA1 expression indicates active proliferation and mitochondrial integrity.

## 4. Conclusions

We demonstrated that VPS35 plays an essential role in regulating cell growth. HEK293 cells lacking VPS35 exhibited markedly reduced proliferation, associated with decreased expression of cell cycle-related proteins and abnormalities in mitochondrial dynamics, suggesting that modulation of VPS35 expression at the molecular level could provide a novel therapeutic approach for controlling unregulated cell proliferation, such as that observed in cancer.

## Data Availability

The original contributions presented in this study are included in the article. Further inquiries can be directed to the corresponding author.

## Abbreviations

The following abbreviations are used in this manuscript:

CDK	Cyclin-dependent kinase
HEK	Human embryonic kidney
KO	Knockout
OPA1	Optic atrophy 1
PARP	Poly(ADP-ribose) polymerase
PBS	Phosphate-buffered saline
TUNEL	Terminal deoxynucleotidyl transferase dUTP nick end labeling
VPS	Vacuolar protein sorting
WT	Wild-type

## References

[1] Brenner S. (1998). Twisted strands. Nature.

[2] Crick F. (1970). Central dogma of molecular biology. Nature.

[3] Hirokawa N., Noda Y., Tanaka Y., Niwa S. (2009). Kinesin superfamily motor proteins and intracellular transport. Nat. Rev. Mol. Cell Biol..

[4] Kamal A., Goldstein L.S. (2002). Principles of cargo attachment to cytoplasmic motor proteins. Curr. Opin. Cell Biol..

[5] Mukadam A.S., Seaman M.N. (2015). Retromer-mediated endosomal protein sorting: The role of unstructured domains. FEBS Lett..

[6] Small S.A., Petsko G.A. (2015). Retromer in Alzheimer disease, Parkinson disease and other neurological disorders. Nat. Rev. Neurosci..

[7] Lucas M., Hierro A. (2017). Retromer. Curr. Biol..

[8] Burd C., Cullen P.J. (2014). Retromer: A master conductor of endosome sorting. Cold Spring Harb. Perspect. Biol..

[9] Lee J.J., Radice G., Perkins C.P., Costantini F. (1992). Identification and characterization of a novel, evolutionarily conserved gene disrupted by the murine H beta 58 embryonic lethal transgene insertion. Development.

[10] Arighi C.N., Hartnell L.M., Aguilar R.C., Haft C.R., Bonifacino J.S. (2004). Role of the mammalian retromer in sorting of the cation-independent mannose 6-phosphate receptor. J. Cell Biol..

[11] Kuchitsu Y., Mukai K., Uematsu R., Takaada Y., Shinojima A., Shindo R., Shoji T., Hamano S., Ogawa E., Sato R. (2023). STING signalling is terminated through ESCRT-dependent microautophagy of vesicles originating from recycling endosomes. Nat. Cell Biol..

[12] Yang Z., Lee K.H., Follett J., Wabitsch M., Hamilton A.N., Collins M.B., Bugarcic A., Teasdale D.R. (2016). Functional characterization of retromer in GLUT4 storage vesicle formation and adipocyte differentiation. FASEB J..

[13] Kim E., Lee J.W., Baek D.C., Lee S.R., Kim M.S., Kim S.H., Imakawa K., Chang K.T. (2008). Identification of novel retromer complexes in the mouse testis. Biochem. Biophys. Res. Commun..

[14] Kim E., Lee Y., Lee H.J., Kim J.S., Song B.S., Huh J.W., Lee S.R., Kim S.U., Kim S.H., Hong Y. (2010). Implication of mouse Vps26b-Vps29-Vps35 retromer complex in sortilin trafficking. Biochem. Biophys. Res. Commun..

[15] Banos-Mateos S., Rojas A.L., Hierro A. (2019). VPS29, a tweak tool of endosomal recycling. Curr. Opin. Cell Biol..

[16] Yu J., Feng H., Sang Q., Li F., Chen M., Yu B., Xu Z., Pan T., Wu X., Hou J. (2023). VPS35 promotes cell proliferation via EGFR recycling and enhances EGFR inhibitors response in gastric cancer. EBioMedicine.

[17] Yang Z., Feng Z., Li Z., Teasdale R.D. (2022). Multifaceted roles of retromer in EGFR trafficking and signaling activation. Cells.

[18] Hong X., Wang T., Du J., Hong Y., Yang C.P., Xiao W., Li Y., Wang M., Sun H., Deng Z.H. (2022). ITRAQ-based quantitatibe proteomic analysis reveals that VPS35 promotes the expression of MCM2-7 genes in HeLa cells. Sci. Rep..

[19] Tang M., Monani U.R. (2021). Glut1 deficiency syndrome: New and emerging insights into a prototypical brain energy failure disorder. Neurosci. Insights.

[20] Liu H.Y., Sun X.J., Xiu S.Y., Zhang X.Y., Wang Z.Q., Gu Y.L., Yi C.X., Liu J.Y., Dai Y.S., Yuan X. (2024). Frizzled receptors (FZDs) in Wnt signaling: Potential therapeutic targets for human cancers. Acta Pharmacol. Sin..

[21] Hayat R., Manzoor M., Hussain A. (2022). Wnt signaling pathway: A comprehensive review. Cell Biol. Int..

[22] Dulski J., Ross O.A., Wszolek Z.K., Adam M.P., Bick S., Feldman J., Mirzaa G.M., Pagon R.A., Wallace S.E., Amemiya A. (1993). VPS35-Related Parkinson Disease. GeneReviews.

[23] Rahman A.A., Morrison B.E. (2019). Contributions of VPS35 Mutations to Parkinson’s Disease. Neuroscience.

[24] Wen L., Tang F.L., Hong Y., Luo S.W., Wang C.L., He W., Shen C., Jung J.U., Xiong F., Lee D. (2011). VPS35 haploinsufficiency increases Alzheimer’s disease neuropathology. J. Cell Biol..

[25] Wang X., Xie Y., Fan X., Wu X., Wang D., Zhu L. (2024). Intermittent hypoxia training enhances Abeta endocytosis by plaque associated microglia via VPS35-dependent TREM2 recycling in murine Alzheimer’s disease. Alzheimers Res. Ther..

[26] Park C., Song Y.K., Kim Y.H., Jung Y., Park Y.H., Song B.S., Eom T., Kim J.S., Kim S.H., Kim J.S. (2019). Development of a New Type of Recombinant Hyaluronidase Using a Hexahistidine; Possibilities and Challenges in Commercialization. J. Microbiol. Biotechnol..

[27] Bang H., Lee S., Jeong P.S., Seol D.W., Son D., Kim Y.H., Song B.S., Sim B.W., Park S., Lee D.M. (2022). Hyaluronidase 6 Does Not Affect Cumulus-Oocyte Complex Dispersal and Male Mice Fertility. Genes.

[28] Kim C., Wang X.D., Jang S., Yu Y. (2023). PARP1 nhibitors induce pyroptosis via caspase 3-mediated gasdermin E cleavage. Biochem. Biophys. Res. Commun..

[29] Tang F.L., Liu W., Hu J.X., Erion J.R., Ye J., Mei L., Xiong W.C. (2015). VPS35 Deficiency or Mutation Causes Dopaminergic Neuronal Loss by Impairing Mitochondrial Fusion and Function. Cell Rep..

[30] Sukumaran P., Conceicao D.V., Sun Y., Ahamad N., Saraiva R.L., Selvaraj S., Singh B.B. (2021). Calcium signaling regulates autophagy and apoptosis. Cells.

[31] Gerdes J., Schwab U., Lemke H., Stein H. (1983). Production of a mouse monoclonal antibody reactive with a human nuclear antigen associated with cell proliferation. Int. J. Cancer.

[32] Lai L., Shin G.Y., Qiu H. (2020). The Role of Cell Cycle Regulators in Cell Survival-Dual Functions of Cyclin-Dependent Kinase 20 and p21(Cip1/Waf1). Int. J. Mol. Sci..

[33] Liao J.Z., Chung H.L., Shih C., Wong K.K.L., Dutta D., Nil Z., Burns C.G., Kanca O., Park Y.J., Zuo Z. (2024). Cdk8/CDK19 promotes mitochondrial fission through Drp1 phosphorylation and can phenotypically suppress pink1 deficiency in Drosophila. Nat. Commun..

[34] Ko H.J., Tsai C.Y., Chiou S.J., Lai Y.L., Wang C.H., Cheng J.T., Chuang T.H., Huang C.F., Kwan A.L., Loh J.K. (2021). The Phosphorylation Status of Drp1-Ser637 by PKA in Mitochondrial Fission Modulates Mitophagy via PINK1/Parkin to Exert Multipolar Spindles Assembly during Mitosis. Biomolecules.

[35] Taguchi N., Ishihara N., Jofuku A., Oka T., Mihara K. (2007). Mitotic phosphorylation of dynamin-related GTPase Drp1 participates in mitochondrial fission. J. Biol. Chem..

[36] Zanfardino P., Amati A., Perrone M., Petruzzella V. (2025). The balance of MFN2 and OPA1 in mitochondrial dynamics, cellular homeostasis and disease. Biomolecules.

[37] Sheridan C., Martin J.S. (2010). Mitochondrial fission/fusion dynamics and apoptosis. Mitochondrion.

